# Appraisal of clinical practice guidelines for management of paediatric type 2 diabetes mellitus using the AGREE II instrument: a systematic review protocol

**DOI:** 10.1186/s13643-016-0288-3

**Published:** 2016-07-13

**Authors:** Dena Zeraatkar, Ahmed Nahari, Pei-Wen Wang, Emily Kearsley, Nicole Falzone, Michael Xu, Laura Banfield, Lehana Thabane, M. Constantine Samaan

**Affiliations:** Department of Pediatrics, McMaster University, 1280 Main Street West, HSC-3A57, Hamilton, Ontario L8S 4K1 Canada; Division of Pediatric Endocrinology, McMaster Children’s Hospital, 1280 Main Street West, HSC-3A57, Hamilton, Ontario L8S 4K1 Canada; Department of Clinical Epidemiology & Biostatistics, McMaster University, Hamilton, Ontario Canada; Health Sciences Library, McMaster University, Hamilton, Ontario Canada; Department of Anesthesia, McMaster University, Hamilton, Ontario Canada; Centre for Evaluation of Medicines, Hamilton, ON Canada; Biostatistics Unit, St. Joseph’s Healthcare Hamilton, Hamilton, Ontario Canada; Department of Pediatrics, King Fahad Central Hospital, Jazan, Saudi Arabia

## Abstract

**Background:**

The prevalence of type 2 diabetes mellitus (T2DM) in children and adolescents is increasing. This has spurred the development and publication of clinical practice guidelines (CPGs) for the management of paediatric T2DM. Given the long-term complications of T2DM, optimal management is important to prevent or delay these complications. However, the quality of published CPGs has not yet been empirically evaluated.

Our objective is to systematically appraise all published CPGs for the management of T2DM in children and adolescents.

**Methods:**

We will identify all published CPGs that address T2DM in children and adolescents through MEDLINE, Embase, CINAHL, Trip, and the National Guideline Clearinghouse and will screen diabetes and paediatric societies and associations’ websites. Search records will be screened in duplicate for inclusion. Grey literature will be covered by systematically searching publications of all relevant diabetes societies and associations and other health organizations for CPGs that meet our inclusion criteria. CPGs deemed eligible for inclusion will be retrieved. Quality assessment will be conducted using the Appraisal of Guidelines for Research and Evaluation II (AGREE II) tool by a team of four appraisers. Scaled scores of the AGREE II will be used to gauge the overall quality of CPGs.

**Discussion:**

The results of this review will be disseminated through presentations at local, national, and international conferences and publication in a peer-reviewed journal. The results of this review can help improve the reporting of future guidelines, inform decisions of policy-makers to endorse CPGs, and affect the choice of guideline use in clinical practice.

**Systematic review registration:**

PROSPERO CRD42016034187

## Introduction

Type 2 diabetes mellitus (T2DM) is a relatively new disease in children, driven mainly by the obesity epidemic [1–3]. While the pathophysiology of paediatric T2DM is not completely understood, the main drivers of its etiopathogenesis are related to a combination of obesity-driven insulin resistance in the skeletal muscle, liver, and adipose tissue [4]. This is coupled with β-cell failure to produce sufficient insulin to overcome insulin resistance due to inflammation, glucotoxicity, and lipotoxicity [5–10].

Paediatric T2DM patients are at risk of secondary comorbidities including dyslipidaemia, hypertension, non-alcoholic fatty liver disease, polycystic ovary syndrome, obstructive sleep apnoea, and psychological problems [11–13]. These comorbidities increase the risk for cardiovascular disease [14]. In addition, T2DM patients can develop acute life-threatening complications including diabetic ketoacidosis and hyperosmolar hyperglycaemic state [15, 16].

Limited therapeutic options exist for children with T2DM. Currently, lifestyle intervention, metformin, and insulin are the only available therapeutic modalities, with other medications undergoing trials to validate their role in children and youth [13, 17, 18].

Due to its relative novelty, there are no natural history data to guide prognosis, and it is possible that children with T2DM will have a shorter lifespan than their non-diabetic counterparts, due to the association of diabetes with cardiovascular and renal causes [19].

In response to this crisis, clinical practice guidelines (CPGs), defined as ‘systematically developed statements to assist practitioner and patient decisions about appropriate healthcare for specific clinical circumstances,’ to guide the care of children and adolescents with T2DM have been developed [20].

The potential for CPGs to enhance the care of children and adolescents with T2DM is dependent on their quality, as well as uptake and adoption in practice. High-quality, evidence-driven CPGs have the potential to improve patient outcomes, due to their ability to standardize the delivery of evidence-based care by a large number of healthcare practitioners. CPGs also have the potential to improve the allocation and utilization of finite healthcare resources and reduce waste.

There is evidence that those CPGs in some health conditions are of variable quality [21–23], and this has the potential to impact care delivery and outcomes.

The purpose of this systematic review is to evaluate the quality of published CPGs for management of T2DM in children and adolescents and to evaluate the changes in their quality over time.

### Research question

Do CPGs of paediatric type 2 diabetes mellitus conform to quality standards based on the AGREE II tool? If so, has the quality of these guidelines changed over time?

### Objectives

#### Primary

The primary objective of this systematic review is to evaluate the quality of published CPGs for the management of T2DM in children and adolescents using the Appraisal of Guidelines for Research and Evaluation II (AGREE II) tool.

#### Secondary

The secondary objective of this systematic review is to determine whether the quality of CPGs that have been revised or updated has improved over time.

## Methods

The protocol is registered with PROSPERO (registration number: CRD42016034187). A PRISMA-P checklist is included to report the various sections under which each item is described (Table 1) [24, 25].

**Table 1:** PRISMA-P checklist for protocol items

Section and topic	Item No	Checklist item	Page No	Line No
ADMINISTRATIVE INFORMATION				
Title:				
Identification	1a	Identify the report as a protocol of a systematic review	1	Title
Update	1b	If the protocol is for an update of a previous systematic review, identify as such	N/A	N/A
Registration	2	If registered, provide the name of the registry (such as PROSPERO) and registration number	2	22
Authors:				
Contact	3a	Provide name, institutional affiliation, e-mail address of all protocol authors; provide physical mailing address of corresponding author	1	-
Contributions	3b	Describe contributions of protocol authors and identify the guarantor of the review	11	209–219
Amendments	4	If the protocol represents an amendment of a previously completed or published protocol, identify as such and list changes; otherwise, state plan for documenting important protocol amendments	N/A	N/A
Support:				
Sources	5a	Indicate sources of financial or other support for the review	11	208
Sponsor	5b	Provide name for the review funder and/or sponsor	11	208
Role of sponsor or funder	5c	Describe roles of funder(s), sponsor(s), and/or institution(s), if any, in developing the protocol	N/A	N/A
INTRODUCTION				
Rationale	6	Describe the rationale for the review in the context of what is already known	3–4	24–55
Objectives	7	Provide an explicit statement of the question(s) the review will address with reference to participants, interventions, comparators, and outcomes (PICO)	4	56–63
METHODS				
Eligibility criteria	8	Specify the study characteristics (such as PICO, study design, setting, time frame) and report characteristics (such as years considered, language, publication status) to be used as criteria for eligibility for the review	4	56–58
			4–5	69–91
Information sources	9	Describe all intended information sources (such as electronic databases, contact with study authors, trial registers or other grey literature sources) with planned dates of coverage	6	92–114
Search strategy	10	Present draft of search strategy to be used for at least one electronic database, including planned limits, such that it could be repeated	Table 2	Table 2
Study records:				
Data management	11a	Describe the mechanism(s) that will be used to manage records and data throughout the review	7	116–117
Selection process	11b	State the process that will be used for selecting studies (such as two independent reviewers) through each phase of the review (that is, screening, eligibility and inclusion in meta-analysis)	7	119–126
Data collection process	11c	Describe planned method of extracting data from reports (such as piloting forms, done independently, in duplicate), any processes for obtaining and confirming data from investigators	7	120–121
			9	163–170
Data items	12	List and define all variables for which data will be sought (such as PICO items, funding sources), any pre-planned data assumptions and simplifications	7	127–134
Outcomes and prioritization	13	List and define all outcomes for which data will be sought, including prioritization of main and additional outcomes, with rationale	8	138–141
Risk of bias in individual studies	14	Describe anticipated methods for assessing risk of bias of individual studies, including whether this will be done at the outcome or study level, or both; state how this information will be used in data synthesis	8–9	153–169
Data synthesis	15a	Describe criteria under which study data will be quantitatively synthesised	9	171–188
	15b	If data are appropriate for quantitative synthesis, describe planned summary measures, methods of handling data and methods of combining data from studies, including any planned exploration of consistency (such as I^2^, Kendall’s τ)	9	171–188
	15c	Describe any proposed additional analyses (such as sensitivity or subgroup analyses, meta-regression)	N/A	N/A
	15d	If quantitative synthesis is not appropriate, describe the type of summary planned	10	184–188
Meta-bias(es)	16	Specify any planned assessment of meta-bias(es) (such as publication bias across studies, selective reporting within studies)	NA	NA
Confidence in cumulative evidence	17	Describe how the strength of the body of evidence will be assessed (such as GRADE)	9	178–179

### Eligibility criteria

Published national or international CPGs from diabetes and paediatric associations that are reported as stand-alone guidelines for T2DM in children and adolescents (2–18 years of age) will be included. In addition, guidelines that address type 1 diabetes mellitus (T1DM) in children and adolescents and dedicate a section on recommendations for managing T2DM in this age group will be screened. CPGs that are targeted to the management of adult T2DM with a separate section with specific recommendations for management of T2DM in children and adolescents will also be included. We will compare the two most recent guidelines from a given agency if available and will not restrict language of publication of the included guidelines.

Unpublished CPGs, CPGs developed for exclusive use within one institution, and CPGs currently under development will be excluded. We will also exclude studies that make recommendations for management of paediatric T2DM but are not CPGs. These include randomized controlled trials (RCTs), including cluster RCTs, controlled (non-randomized) clinical trials, and case-control, prospective and retrospective cohort, and cross-sectional studies in T2DM. We will also exclude case reports, pilot and feasibility studies, and conference abstracts and posters.

We will also exclude guidelines that deal with drug-induced diabetes including steroids, antipsychotic medications, and immunomodulatory therapies or guidelines that deal with genetic forms of diabetes, e.g. maturity-onset diabetes of the young (MODY).

The primary outcome is the evaluation of the quality of the guidelines by calculating the AGREE II tool score for treatment of T2DM in children and adolescents.

The secondary outcome is to determine whether the quality of CPGs that have been revised or updated has improved over time.

### Data sources and search strategy

The search strategy will be developed in consultation with a health sciences librarian with expertise in systematic reviews. We will search databases including MEDLINE and Embase through the Ovid interface, Cumulative Index to Nursing and Allied Health Literature (CINAHL), Trip, and the National Guideline Clearinghouse (guideline.gov). The search will be conducted from the inception of each database up to and including January 31, 2016.

The search will be tailored to the capabilities of each database. Medical Subject Headings (MeSH) and keywords relating to (1) CPGs, (2) diabetes, and (3) children and/or adolescents will be combined using Boolean search operators. The search will not be limited to T2DM in order to capture guidelines that cover both T1DM and T2DM.

The search will be limited by age group and publication type. The age range will be specified as 2–18 years, effectively excluding references on gestational diabetes or congenital forms of diabetes. The publication type will be limited to guidelines. Table 2 presents a sample search, which will be run on MEDLINE. The search strategy may be revised to enhance sensitivity and specificity.

**Table 2:** Sample search conducted on MEDLINE using the OVID interface

#	Searches
1	guideline/ or practice guideline/
2	guideline*.ti,ab,kf.
3	guideline.pt.
4	Consensus Development Conference/
5	consensus statement*.ti,ab,kf.
6	or/1–5
7	diabetes mellitus/or exp diabetes mellitus, type 1/or exp diabetes mellitus, type 2/
8	diabet*.ti,ab,kf.
9	IDDM.ti,ab,kf.
10	NIDDM.ti,ab,kf.
11	(T1DM or T2DM or T1D or T2D).ti,ab,kf.
12	((noninsulin or non insulin or insulin) adj2 depend*).ti,ab,kf.
13	or/7–12
14	adolescent/ or child/ or child, preschool/
15	child*.ti,ab,kf.
16	adolescen*.ti,ab,kf.
17	teen*.ti,ab,kf.
18	youth*.ti,ab,kf.
19	p?ediatric*.ti,ab,kf
20	Pediatrics/
21	or/14–20
15	6 and 13 and 21

To identify guidelines published outside of indexed journals, our search of key databases will be supplemented with a search of the grey literature. This will be covered by systematically searching publications of all relevant diabetes societies and associations and other health organizations for CPGs that meet our inclusion criteria.

The world will be divided into the Americas (North and South), Europe, Asia (including Australasia and Oceania), and Africa. Four reviewers will be tasked with systematically searching online for relevant CPGs published by diabetes societies and associations and other medical and health organizations in their assigned regions.

### Data management

Search results will be uploaded to a private group on Mendeley (https://www.mendeley.com), a reference management tool. Exact duplicates will be removed.

### Data selection process

Two independent reviewers will screen titles and abstracts of records on Mendeley, and those deemed eligible for inclusion will be retrieved and uploaded onto an external database. Retrieved eligible records will undergo duplicate full-text screening for inclusion. Reasons for exclusion will be documented and reported. Disagreements between reviewers will be resolved by discussion or by consulting an expert paediatric endocrinologist (MCS). The team has been educated on the subject area by an expert clinician and researcher (MCS) to ensure familiarity with the research field.

### Data items

For data extraction protocol, we will include the title, authors, issuing society/association, country, year of publication or update, criteria and frequency of screening for T2DM, screening test(s) recommended, and diagnostic criteria. We will also include recommendation of healthcare delivery including personnel needed and recommendations on physical activity, diet, screen time, psychosocial issues, medical and surgical therapies, target HbA1c, and screening methods and frequency for comorbidities including nephropathy, retinopathy, hypertension, dyslipidemia, non-alcoholic fatty liver disease, neuropathy, and polycystic ovary syndrome.

### Outcomes and prioritization

The primary outcome is the score of the AGREE II guideline. The secondary outcome is the demonstration of improvement in score over time for those agencies that have updated versions of their guidelines.

### Quality appraisal

CPGs deemed eligible for inclusion will be appraised using the AGREE II [26]. The AGREE II is a generic instrument developed for quality evaluation of health-related CPGs. It evaluates the process of guideline development and reporting across six domains (scope and purpose, stakeholder involvement, rigour of development, clarity of presentation, editorial independence, and applicability) using a seven-point Likert scale (whereby seven indicates the highest quality). The instrument also contains two additional global items. The first item is on the overall assessment of the quality of the guideline on a seven-point Likert scale, and the second item is on whether the guideline can be recommended for use, which is assessed on an ordinal scale of ‘yes’, ‘yes with modifications’, and ‘no’.

The AGREE II instrument was chosen due to its already widespread use and validation [26–28]. The instrument can be used by researchers, clinicians, and policy-makers with varying experiences in the development and use of CPGs [26]. In user-testing, the instrument has shown acceptable reliability and construct validity, with statistically significant (*p* < 0.05) differences found in the scores of high- and low-quality guidelines of all but three of the 23 items [27, 28]. The number of appraisers required for an inter-rater reliability of 0.7 ranges from two to five across domains [26]. Although only two of the domains achieved an alpha value that met conventionally accepted standards for internal consistency (Cronbach’s alpha ≥ 0.8), this could be attributed to the small number of items in each domain (range 2–8).

A team of four appraisers will perform quality assessment; this is the preferred number of appraisers necessary to reach acceptable inter-rater reliability (intra-class correlation coefficient (ICC) ≥ 0.7) on the AGREE II [26, 27]. Prior to appraisal, appraisers will complete the AGREE II Online Training Tool (http://www.agreetrust.org/resource-centre/agree-ii-training-tools/) and will participate in at least three rounds of calibration with a sample of relevant CPGs of varying qualities. Additional rounds of calibration will be completed until an inter-rater reliability of 0.7 is achieved. All appraisals will be conducted using the online My AGREE PLUS platform (http://www.agreetrust.org/resource-centre/agree-plus/).

### Data synthesis and analysis

All statistical analyses will be conducted using SPSS Statistics Version 22.

**Fig. 1:**
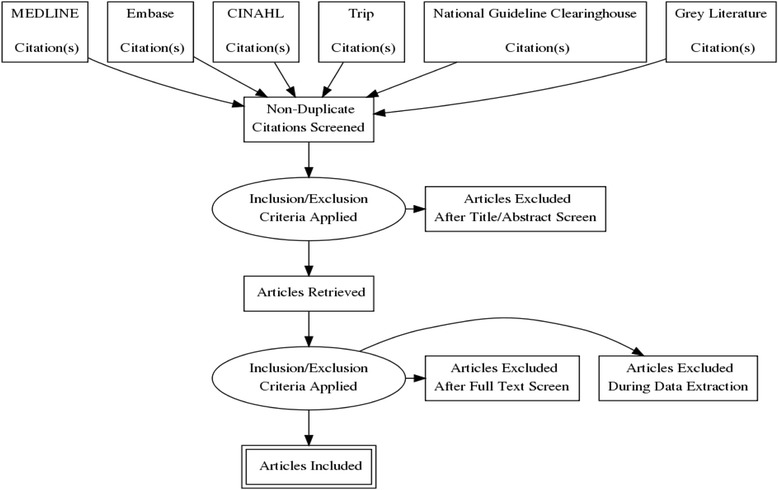
Flow chart for steps taken using the AGREE II tool for evaluation of available paediatric type 2 diabetes guidelines

A flow diagram (Fig. 1) will be constructed to illustrate the flow of citations through the course of this systematic review.

Agreement regarding CPG eligibility between reviewers will be reported as Cohen’s kappa. The target level of agreement will be set as 0.8, indicating strong agreement between reviewers.

Descriptive statistics will be generated and tabulated to summarize the characteristics of CPGs deemed eligible for inclusion. For each CPG, a quality score will be calculated for each of the six domains of AGREE II, using the formula presented in the AGREE II User’s Manual [26]. Briefly, domain scores will be calculated by summing up all the appraisers’ scores of the individual items in a domain and by scaling the total as a percentage of the maximum possible score for that domain. This calculation is automatically generated on the My AGREE PLUS platform. Inter-rater reliability between appraisers for each domain of the AGREE II will be presented as ICCs, with a target ICC of 0.7 for all domains. The overall quality of the included CPGs in each domain of the AGREE II will be presented in a table using summery statistics (mean ± SD) for normally distributed data and medians (minimum-maximum) for skewed distributions. As the six domains of the AGREE II are independent, domain quality scores will not be aggregated.

### Dissemination

The results of this review will be presented at local, national, and international conferences and published in a peer-reviewed journal.

## Discussion

With the increasing prevalence of T2DM in children and adolescents and the burden of living with the disease, there is a critical need to ensure that the evidence used in management of these children is of high quality. In this systematic review, we will investigate the quality of existing CPGs designed to manage paediatric T2DM.

Our proposed review can aid clinicians who wish to enhance the care of children and/or adolescents with T2DM by adopting CPGs in their practice, guideline developers who wish to create better quality CPGs or improve existing ones, researchers who wish to identify knowledge gaps, and policy-makers looking to endorse CPGs. The collation of this evidence will also help to identify gaps in the available CPGs.

The extensive search strategy covering both indexed and grey literature, use of multiple appraisers who will complete training and calibration to assess the quality of CPGs, and application of the AGREE II instrument, which has established validity and reliability [27, 28], are all strengths of this review and may help the delivery of better care.
